# Lateral Heterotaxy Syndrome in a Newborn Caucasian Male

**DOI:** 10.7759/cureus.11205

**Published:** 2020-10-27

**Authors:** Zachary A Koenig, Alex Verhoeven, David Rosen, Ashley B Petrone

**Affiliations:** 1 Medicine, West Virginia University School of Medicine, Morgantown, USA; 2 Pediatrics, West Virginia University School of Medicine, Morgantown, USA; 3 Anesthesiology, West Virginia University School of Medicine, Morgantown, USA; 4 Pathology, Anatomy, Laboratory Medicine, West Virginia University School of Medicine, Morgantown, USA

**Keywords:** heterotaxy syndrome, ivemark syndrome, visceral situs malposition, asplenia with cardiac abnormalities, isomerism

## Abstract

Heterotaxy syndrome is a varied spectrum of rearrangements of thoracic and abdominal organs that present many unique complications. Among all congenital deformities, heterotaxy syndrome is rare although this is likely an underestimate without routine imaging due to the benign nature of some defects. Numerous genes have been identified that play a role in its pathogenesis, and it has been hypothesized that heterotaxy syndrome is a consequence of both genetic and environmental impacts on the body axis. This case report also demonstrates the fundamental role of cardiac catheterization and imaging in further specifying the subtype of heterotaxy. Furthermore, it highlights the inconsistency of laterality with functional asplenia, visceral situs ambiguus, double-outlet right ventricle, and a left-sided inferior vena cava apart from other anomalies in a newborn male.

## Introduction

Heterotaxy syndrome, or situs ambiguus, is a constellation of thoracoabdominal congenital irregularities involving organ rearrangements across the body axis. Situs ambiguus is a constellation of findings without a definitive archetype. It differs from situs inversus, which refers to the total mirror image of organs within the body cavity, and situs solitus, which refers to typical abdominal organ positioning. Rather, heterotaxy syndrome is a rarer, intermediate form of visceral malposition referring to variability in the laterality of individual organs instead of the whole compartment [1].

The complex is often subdivided into left and right atrial isomerism, which conventionally refers to the anatomy of the designated side presenting bilaterally [2]. Rather than having invariable pathognomonic features, this nomenclature tends to refer to the general pattern found within the abdominal and thoracic compartments. More simply put, it often describes the duplication of same-side structures and absence of those on the opposite side. As such, left atrial isomerism is typically associated with polysplenia, biliary atresia, bilateral superior vena cavae, and bilateral hyparterial and bilobed lungs, whereas patients with right isomerism frequently present with asplenia, pulmonary stenosis or atresia, dextrocardia, bilateral eparterial and trilobed lungs [2]. Asplenia, as is often seen in right atrial isomerism, predisposes to recurrent infections from encapsulated bacteria [3,4]. Left atrial isomerism and its associated polysplenia still predisposes to infection because multiple spleens are typically dysfunctional [3,4]. Common to both types of isomerism are intestinal rotational abnormalities, which can result in acute midgut volvulus [5].

In addition to the findings previously mentioned, heterotaxy syndrome exhibits a myriad of cardiac manifestations. Right atrial isomerism is frequently connected with pulmonary valvar stenosis or atresia, obstruction of pulmonary veins with anomalous pulmonary venous return, and septation defects with a predisposition for univentricular anomalies [1,6]. The cardiac anomalies seen in left atrial isomerism are more wide-ranging and are more likely to be subclinical. However, the structural cardiac anomalies in left atrial isomerism predispose to electrophysiological phenomena of which complete heart block is the predominant form and associated with significant morbidity and mortality [1,6].

## Case presentation

A male infant was born at 37 weeks and three days gestation following induction of labor due to fetal growth restriction. The newborn was suspected to be affected by maternal exposure to tobacco and Subutex. There was no known family history of heterotaxy syndrome or situs spectrum disorders. Fetal echocardiogram had been performed prior to delivery and demonstrated complicated cardiovascular findings in line with heterotaxy syndrome. No karyotype, cytogenetic, or fluorescent in-situ hybridization (FISH) anomalies were identified via prenatal genetic testing. His genetic heterotaxy panel (microarray) also did not reveal any known mutations. Upon delivery, he showed marked cyanosis and required cardiopulmonary resuscitation and continuous positive airway pressure (CPAP). His oxygen saturation measured at 75-80% after initial attempts at resuscitation. He was ultimately intubated and placed on mechanical ventilation. Postnatal imaging was performed to clarify the suspected fetal diagnosis.

**Figure 1:**
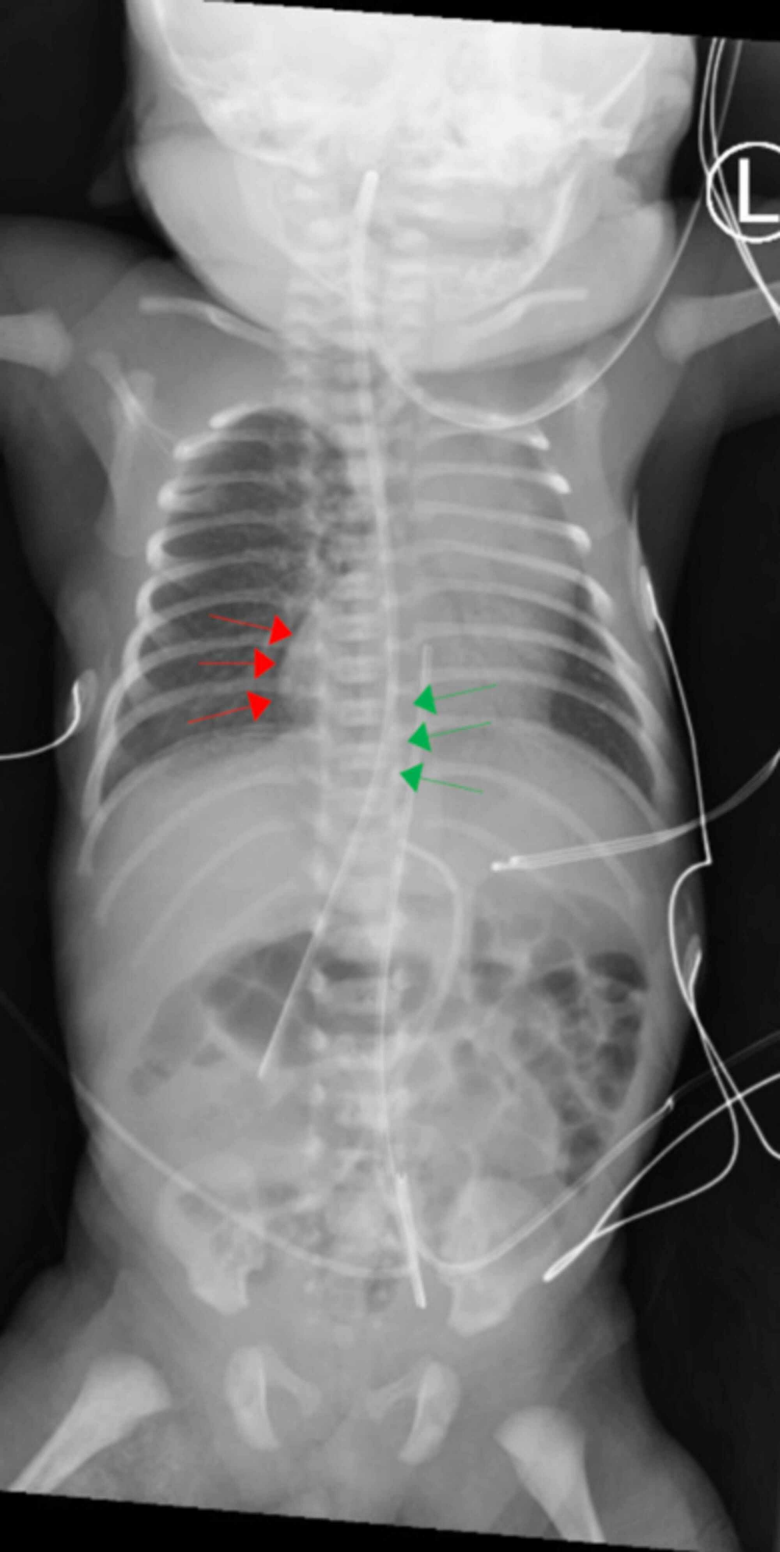
Mobile chest and abdominal X-ray indicate entrance of the gastric tube (green) to the right side of the patient’s body. The red line indicates the apex of the heart on the patient's right side. A gastric air bubble is apparent on the patient’s anatomical right side. Also the liver appears midline. There is no evidence of gut malrotation.

Chest and abdominal x-ray confirmed dextrocardia with a right-sided heart apex. The gastric air bubble was apparent on the right with a nasogastric tube that was positioned in the right upper quadrant. The bowel gas pattern demonstrated enlargement of the gastric air bubble and absence of bowel gas projecting over the right abdomen, concerning for possible malrotation (Figure 1). Follow-up with barium fluoroscopy showed that the esophagus, duodenum, and small bowel were normal, and the small bowel was patent without evidence of malrotation. Ultrasound revealed a structurally normal, horizontally oriented liver in the right upper quadrant that stretched across the midline (Figure 1). The spleen appeared normal in size and echogenicity. 

Respiratory cultures were positive for unspecified gram-positive cocci and lactose-fermenting gram-negative rods at post-natal day 10. Given the association of functional asplenia and heterotaxy, infectious disease was consulted. Peripheral blood smears were positive for schistocytes and Howell-Jolly bodies, confirming splenic dysfunction. As a result, he would later require prophylactic amoxicillin to cover common encapsulated organisms.

**Figure 2:**
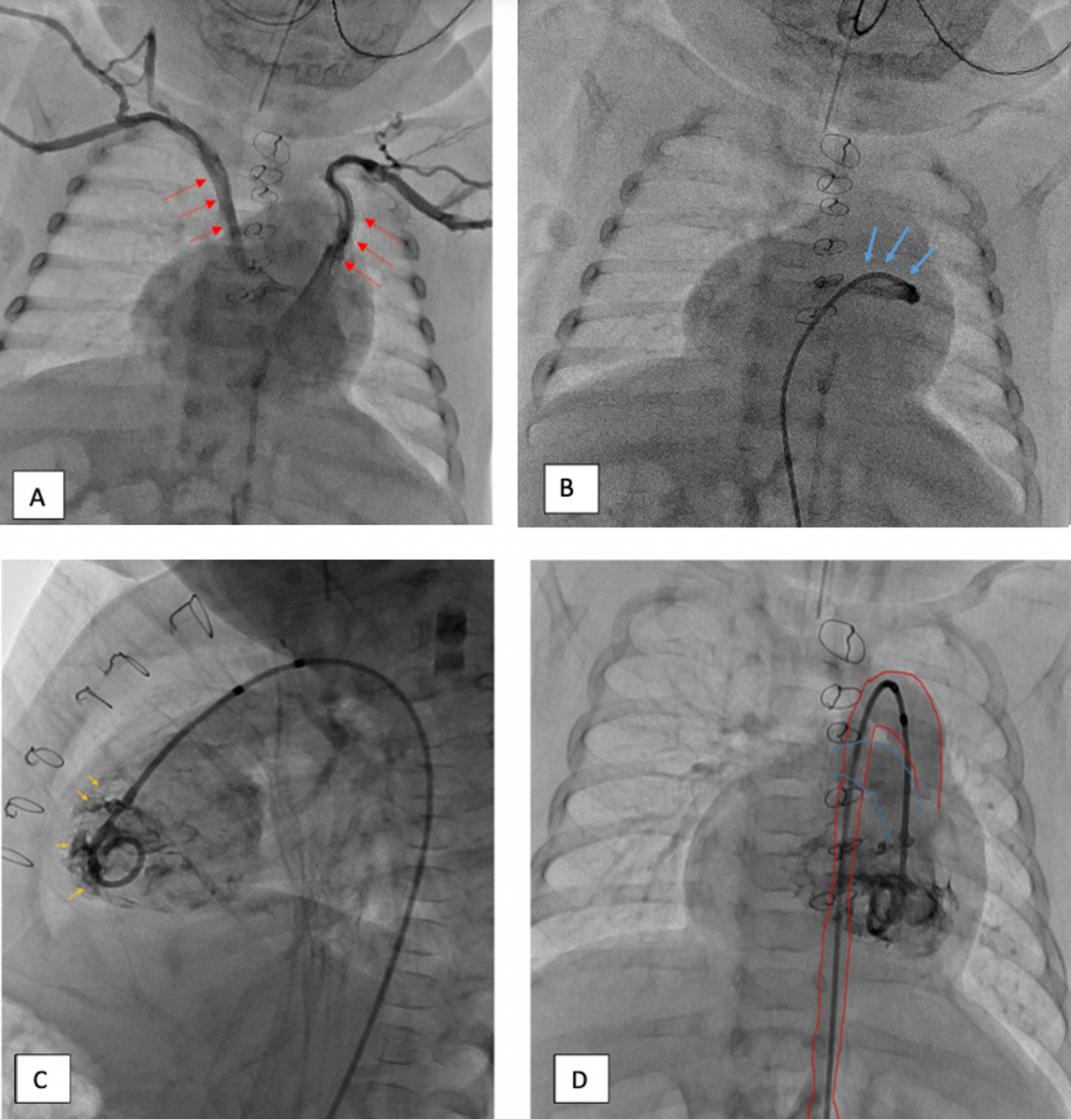
A: Bilateral superior vena cava (red) drained into the left side of the common atrium. B: Anomalous pulmonary venous drainage (blue) into the right side of the common atrium. C: Aorta originating from the patient's left-sided ventricle. Trabeculated walls (yellow) indicates that the patient's left-sided ventricle is an anatomical right ventricle (yellow arrows). D: Mal-posed aorta (red) and main pulmonary artery (blue) originating from the patient’s left-sided ventricle.

**Figure 3:**
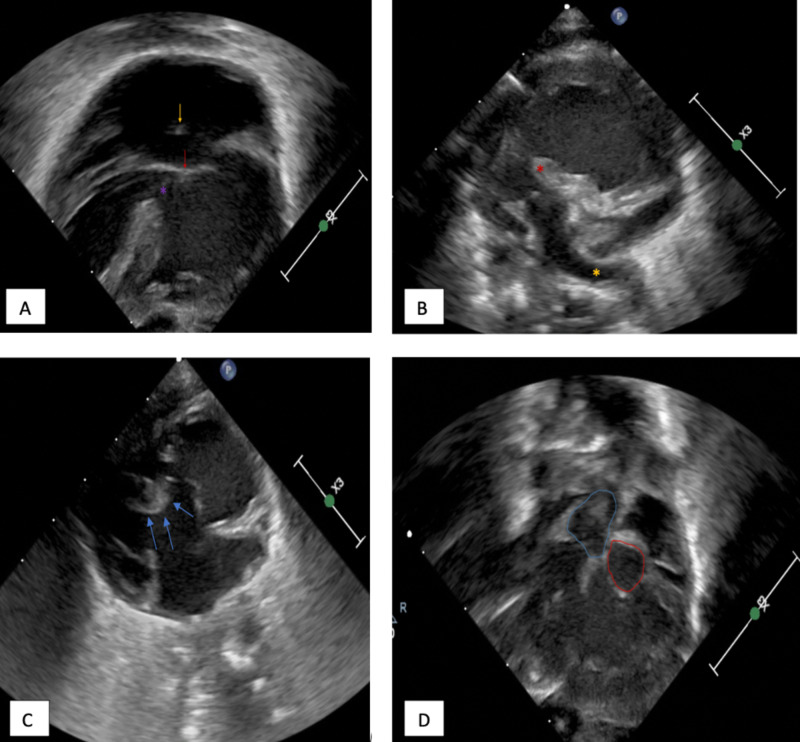
A: Ultrasonography apical 4-chamber view of the heart. There is a common atrium with small intact septum (yellow arrow), common atrioventricular valve (red arrow), and ventricular septal defect (purple asterisk). B: The main pulmonary artery originates from the left ventricle with the left pulmonary artery clearly visualized (yellow asterisk). The infundibulum is labelled with red asterisk. Sub-pulmonary stenosis is also noted. C: Visualization of the patient’s left-sided ventricle. The attachment of valve leaflet to ventricular septum (blue arrow) is further confirmation of right ventricle. D: Ultrasonography right ventricular outflow tract view. There is a double outlet right ventricle, which gives rise to the pulmonary trunk and aorta.

Transthoracic echocardiography and angiography helped clarify the conglomeration complex congenital heart irregularities. There were bilateral superior vena cava draining into the left-sided right atrium (Figure 2A). The inferior vena cava entered the thoracic cavity in the midline before crossing over and ultimately draining into the right-sided atrium. All four pulmonary veins drained into the right-sided right atrium (Figure 2B). There were two side-by-side right atria which both appeared to be anatomically right atria. There was a 6.8 mm septum primum and 8.3 mm septum secundum defect, which functionally resulted in a single common atrium with unrestricted flow across the atrial septum (Figure 3A). There was a balanced, common atrioventricular valve. The anatomic right ventricle was on the patient’s left, and the smooth-walled anatomic left ventricle on the patient’s right (Figure 2C, 3C). The anatomic right ventricle gave rise to both the aorta and main pulmonary artery thus constituting a double-outlet right ventricle (Figure 3D). There was severe sub-pulmonary valve stenosis with no appreciable regurgitation (Figure 3B). There was a tri-leaflet aortic valve without stenosis or regurgitation. The aortic and pulmonary outflow tracts were separated by a prominent conal septum, resulting in significant subpulmonic stenosis. Additionally, the aorta and main pulmonary artery were malposed with the main pulmonary artery located to the right of the ascending aorta (Figure 2D). The main pulmonary artery was slightly hypoplastic, and there was no stenosis of either the left or right branch pulmonary arteries. 

## Discussion

The majority of cases of heterotaxy syndrome do not show any symptoms [7]. On the other end of the spectrum, some patients with heterotaxy syndrome harbor severe cardiac anomalies along with the typical visceral organ axis deviations. Left atrial isomerism is similar to right atrial isomerism, but the prognosis is much better because of the overall less severe associated cardiac abnormalities. Specifically, heterotaxy patients with asplenia have a prognosis greater than 85%, whereas those with polysplenia have a prognosis of greater than 50% [8]. The mainstay of treatment for heterotaxy patients with complex cardiac lesions is heart surgery and catheterization to repair valvular and chamber defects [1,6]. Here, we discuss the classification of heterotaxy syndrome showing discordance of the cardiac apex and abdominal viscera, double outlet right ventricle with atrioventricular canal, bilateral superior vena cava, and anomalous pulmonary venous drainage seen in a newborn Caucasian male.

**Figure 4:**
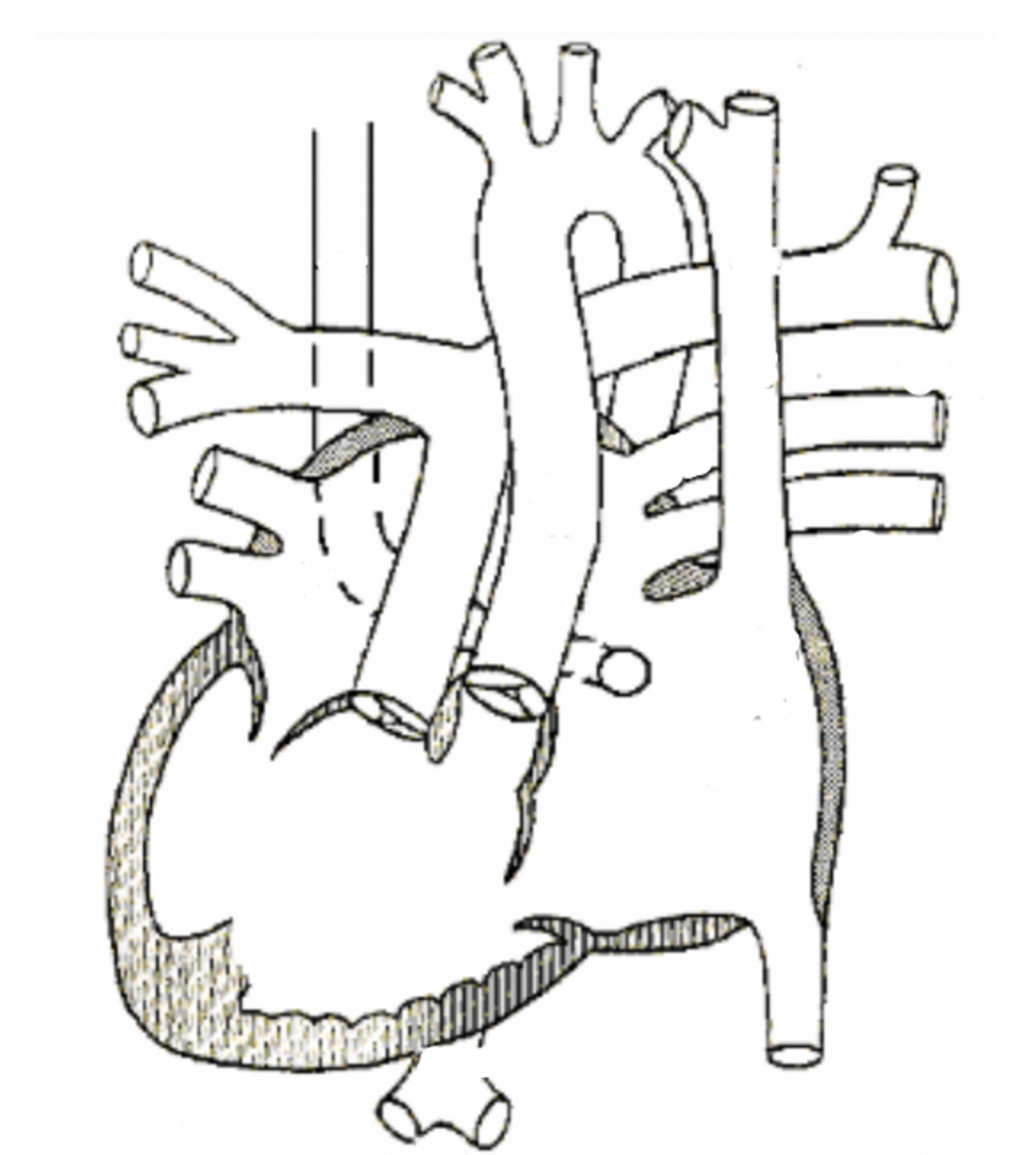
Schematic of observed cardiac malformations. It demonstrates the double-outlet right ventricle, complete atrioventricular canal, and anomalous pulmonary venous drainage.

The individual described in the case report presented with a constellation of findings accordant with the diagnosis of heterotaxy syndrome (Figure 4). However, the exact form of heterotaxy syndrome is hard to discern due to incongruent features. For example, double-outlet right ventricle, transposition of the great vessels, and pulmonary and sub-pulmonary stenosis point towards a diagnosis of right atrial isomerism [1,6,9]. On the other hand, the balanced common atrioventricular canal defect and persistent left superior vena cava hint at left atrial isomerism [10-12]. It is important to highlight the pertinent negatives in this case, including lack of bilateral structures with right or left morphologies, and inapparent abnormal ventricular hypertrophy, anomalous pulmonary venous drainage, and coarctation of the aorta. The direction of the cardiac apex in the thorax is not strongly associated with isomerism of any particular side [1]. 

## Conclusions

This patient’s heterotaxy syndrome has components which appear consistent with both right and left atrial isomerism. Numerous studies have reported common anatomic features associated with each form of isomerism, but the etiology and features of the disease remains an active area of research. Using the example presented above, it is likely that heterotaxy syndrome represents a spectrum of lateralization sub-types depending on the specific etiology of the dysfunctional laterality of each patient. Future work should aim to develop more nuanced nomenclature to better capture the spectrum of disease seen in heterotaxy.
